# Patient-derived xenografts of triple-negative breast cancer reproduce molecular features of patient tumors and respond to mTOR inhibition

**DOI:** 10.1186/bcr3640

**Published:** 2014-04-07

**Authors:** Haiyu Zhang, Adam L Cohen, Sujatha Krishnakumar, Irene L Wapnir, Selvaraju Veeriah, Glenn Deng, Marc A Coram, Caroline M Piskun, Teri A Longacre, Michael Herrler, Daniel O Frimannsson, Melinda L Telli, Frederick M Dirbas, AC Matin, Shanaz H Dairkee, Banafshe Larijani, Gennadi V Glinsky, Andrea H Bild, Stefanie S Jeffrey

**Affiliations:** 1 Division of Surgical Oncology, Stanford University School of Medicine, Stanford, CA 94305, USA; 2 Division of Oncology, Huntsman Cancer Institute, University of Utah, Salt Lake City UT 84112, USA; 3 Stanford Genome Technology Center, Stanford University School of Medicine, Palo Alto, CA 94304, USA; 4 Cell Biophysics Laboratory, London Research Institute, Cancer Research UK, London, UK; 5 College of Life Science and Chemistry, Wuhan Donghu University, Wuhan, Hubei, China; 6 Department of Health Research and Policy (Biostatistics), Stanford University School of Medicine, Stanford, CA 94305, USA; 7 Department of Medical Sciences, School of Veterinary Medicine, University of Wisconsin-Madison, Madison, WI 53706, USA; 8 Department of Pathology, Stanford University School of Medicine, Stanford, CA 94305, USA; 9 Life Technologies Corporation, Department of Medical Sciences, Foster City, CA 94404, USA; 10 Department of Microbiology and Immunology, Stanford University School of Medicine, Stanford, CA 94305, USA; 11 Division of Medical Oncology, Stanford University School of Medicine, Stanford, CA 94305, USA; 12 California Pacific Medical Center Research Institute, San Francisco, CA 94107, USA; 13 Sanford-Burnham Medical Research Institute, La Jolla, CA 92037, USA; 14 Department of Pharmacology and Toxicology, University of Utah, Salt Lake City, UT 84112, USA

## Abstract

**Introduction:**

Triple-negative breast cancer (TNBC) is aggressive and lacks targeted therapies. Phosphatidylinositide 3-kinase (PI3K)/mammalian target of rapamycin (mTOR) pathways are frequently activated in TNBC patient tumors at the genome, gene expression and protein levels, and mTOR inhibitors have been shown to inhibit growth in TNBC cell lines. We describe a panel of patient-derived xenografts representing multiple TNBC subtypes and use them to test preclinical drug efficacy of two mTOR inhibitors, sirolimus (rapamycin) and temsirolimus (CCI-779).

**Methods:**

We generated a panel of seven patient-derived orthotopic xenografts from six primary TNBC tumors and one metastasis. Patient tumors and corresponding xenografts were compared by histology, immunohistochemistry, array comparative genomic hybridization (aCGH) and phosphatidylinositol-4,5-bisphosphate 3-kinase, catalytic subunit alpha (PIK3CA) sequencing; TNBC subtypes were determined. Using a previously published logistic regression approach, we generated a rapamycin response signature from Connectivity Map gene expression data and used it to predict rapamycin sensitivity in 1,401 human breast cancers of different intrinsic subtypes, prompting *in vivo* testing of mTOR inhibitors and doxorubicin in our TNBC xenografts.

**Results:**

Patient-derived xenografts recapitulated histology, biomarker expression and global genomic features of patient tumors. Two primary tumors had PIK3CA coding mutations, and five of six primary tumors showed flanking intron single nucleotide polymorphisms (SNPs) with conservation of sequence variations between primary tumors and xenografts, even on subsequent xenograft passages. Gene expression profiling showed that our models represent at least four of six TNBC subtypes. The rapamycin response signature predicted sensitivity for 94% of basal-like breast cancers in a large dataset. Drug testing of mTOR inhibitors in our xenografts showed 77 to 99% growth inhibition, significantly more than doxorubicin; protein phosphorylation studies indicated constitutive activation of the mTOR pathway that decreased with treatment. However, no tumor was completely eradicated.

**Conclusions:**

A panel of patient-derived xenograft models covering a spectrum of TNBC subtypes was generated that histologically and genomically matched original patient tumors. Consistent with *in silico* predictions, mTOR inhibitor testing in our TNBC xenografts showed significant tumor growth inhibition in all, suggesting that mTOR inhibitors can be effective in TNBC, but will require use with additional therapies, warranting investigation of optimal drug combinations.

## Introduction

Triple-negative breast cancers (TNBCs), which lack expression of estrogen receptor (ER), progesterone receptor (PR) and human epidermal growth factor receptor 2 (HER2), account for approximately 10 to 17% of all breast cancers [1-3] and are associated with relatively poor clinical outcomes. About 70 to 80% of TNBCs comprise the basal-like breast cancer (BLBC) intrinsic subtype as defined by gene expression profiling [4-6], although more recently, TNBCs have been further subclassified into six subtypes distinguished by gene ontologies and gene expression patterns [7,8]. The lack of targeted therapies for this aggressive breast cancer subtype is a key treatment issue and testing new therapeutic regimens is clinically important.

The mammalian target of rapamycin (mTOR) is a key downstream regulator of the phosphatidylinositide 3-kinase (PI3K) pathway, one of the most commonly activated signaling pathways in cancer [9,10]. mTOR exists in two complexes, mTORC1 and mTORC2. mTORC2 is less well understood but has been shown to regulate cell proliferation and cytoskeletal organization [11,12]. PI3K/mTORC1 is frequently activated in human cancers by gain-of-function mutations and amplifications of its upstream activators - such as epidermal growth factor receptor (EGFR), HER2 [13], PI3K or protein kinase B (AKT) - and by the loss of its suppressors, such as phosphatase and tensin homologue (PTEN) [14], inositol polyphosphate-4-phosphatase, type II (INPP4B) [15], or the tuberous sclerosis complex (TSC), mediated by the tumor suppressor genes, *TSC1* and *TSC2*[16,17]. Activated mTORC1, an evolutionarily conserved serine/threonine kinase, will phosphorylate downstream proteins, such as p70 ribosomal S6 kinase 1 (S6K1) [18] and eukaryotic translation initiation factor 4E binding protein 1 (4EBP1) [19], to regulate protein synthesis, ribosome biogenesis and autophagy that contribute to cell proliferation, differentiation and survival [17,20-22]. Activation of the AKT/mTOR pathway is a poor prognostic factor for many types of cancers, including breast cancer [23-27].

Rapamycin (sirolimus) is a specific allosteric inhibitor of mTOR and is the active form of rapamycin analogs. The rapamycin analogs CCI-779 (temsirolimus) and RAD001 (everolimus) are approved for the clinical treatment of advanced renal cell carcinoma [28], progressive neuroendocrine tumors of pancreatic origin [29], subependymal giant cell astrocytoma associated with tuberous sclerosis [30], and more recently for postmenopausal women with advanced hormone receptor-positive, HER2-negative breast cancer in combination with the aromatase inhibitor exemestane [31]. Pertinent for other types of breast cancer, increasing lines of evidence indicate that the PI3K/mTOR pathway is activated in TNBCs and/or BLBCs at the genetic, gene expression and protein levels [14,32-37]. mTOR inhibitors show growth inhibition of TNBC cell lines in both *in vitro* and *in vivo* preclinical studies [14,26,33,38]. *PIK3CA* mutations have been shown to be associated with mTOR inhibitor sensitivity in both cell lines and clinical studies [39-41]. mTOR inhibitors are among the therapeutic agents being actively investigated in clinical trials in patients with TNBC [42-44], and recently, a phase II trial evaluating a combination of everolimus and carboplatin showed a clinical benefit rate of 36% in metastatic TNBC patients [42].

In contrast to previous *in vivo* preclinical drug testing studies using xenografts derived from established breast cancer cell lines, we were interested in determining preclinical drug efficacy in patient-derived TNBC orthotopic xenograft models generated from human tumors obtained fresh from the operating room. Personalized tumorgraft models, also called “avatars”, propagated using patient-derived tumors have shown some success when used to guide clinical treatment in patients with advanced cancer [45,46].

We generated a panel of seven patient-derived orthotopic xenograft models of primary and metastatic TNBC and showed that these models recapitulated histologic and molecular features of the patients’ tumors from which they were derived. We used the Connectivity Map, a compendium of genome-wide transcriptional data from cultured human cells treated with bioactive small molecules, to determine a rapamycin response signature. Applying this signature to large breast cancer datasets stratified into intrinsic breast cancer subtypes, we predicted that most BLBCs would show some sensitivity to rapamycin. We then proceeded with *in vivo* drug testing of two mTOR inhibitors, sirolimus and temsirolimus, in our patient-derived TNBC models, which demonstrated significant growth inhibition by both drugs. However, while growth inhibition was very impressive for all TNBC xenografts, none had complete tumor ablation. Our results strongly support the use of mTOR inhibitors as part of combined therapy for TNBC in preclinical and clinical trials and suggest the need for further investigations into appropriate drug combinations.

## Materials and methods

### Establishment of patient-derived orthotopic xenografts

Both the Stanford University Research Compliance Office’s Human Subjects Research and IRB Panel and Stanford’s Administrative Panel on Laboratory Animal Care (APLAC) approved this study. After obtaining informed written patient consent, breast cancer tissues were obtained fresh from operating rooms at Stanford Hospital and Clinics. In six cases of TNBC (SUTI097, SUTI103, SUTI110, SUTI151, SUTI319, SUTI368), fresh tumor tissue was sterilely obtained from primary breast cancer tissue that was undergoing surgical excision, and in one case (SUTI151M), the tumor tissue was taken fresh from a soft tissue TNBC metastasis to the quadriceps muscle in the thigh that was undergoing biopsy (SUTI151M is from the same patient who had months earlier donated a piece of her primary breast tumor SUTI151). Portions were frozen or placed in formalin and embedded in paraffin for later analyses. Fresh tumor tissue was kept on ice in RPMI 1640 medium supplemented with penicillin/streptomycin and 10% heat inactivated FBS (Invitrogen-Life Technologies, Carlsbad, CA, USA) for transport, minced into one to two millimeter fragments, then sterilely and orthotopically transplanted into the number two mammary fat pads of 5 to 10 female NOD SCID mice (NOD.CB17-*Prkdc*^*scid*^/J, Jackson Laboratory West, Sacramento, CA, USA). Briefly, the mice were anesthetized by inhalation of 1 to 3% isoflurane, their hair was clipped, and their skin sterilized with povidone-iodine and alcohol. A small skin incision was made in the lateral flank and minced tumor chunks were mixed with LDEV-free Matrigel (BD Biosciences, San Jose, CA, USA) and implanted into the mammary fat pad by trochar insertion. The incision site was closed with Vetbond tissue adhesive (3 M, St. Paul, MN, USA). Mice were maintained in pathogen-free animal housing. The established xenografts were subsequently passaged from mouse to mouse to expand xenograft numbers; xenograft tumors were also stored frozen in FBS containing 10% dimethyl sulfoxide (DMSO, EMD Chemicals Inc., Billerica, MA, USA) solution for future engraftment. Xenograft tumor tissue was frozen on dry ice for RNA isolation and microarray analysis and for subsequent protein analyses. Tumor fragments were also fixed in phosphate buffered saline with 10% formalin (Sigma-Aldrich, St. Louis, MO, USA) for histological studies. All animal care was performed in accordance with Stanford University and IACUC guidelines.

### Immunohistochemistry

Formalin-fixed, paraffin-embedded tissue sections of patient or xenograft tumors were cut into 4 μm sections, deparaffinized in xylene, rinsed in ethanol and rehydrated. Staining was performed using the Ventana XT platform and internal antigen retrieval CC1 standard. The antibodies used were rabbit monoclonal antibodies for ERα (clone SP1, 1:25 dilution, Thermo Scientific, Fremont, CA, USA) and PR (clone 1E2, ready to use, Roche-Ventana Medical Systems, Inc., Tucson, AZ, USA). The universal secondary protocol and the DAB MAP kit (Ventana Medical Systems, Inc., Tucson, AZ, USA) were used to detect and amplify the signal. Both biomarkers were scored using a three-tier system: 0 = negative, 1 = weak, and 2 = strong, respectively defined as <1%, 1% to 50%, and ≥50% of tumor cell nuclei staining positively. HER2 protein expression was performed and interpreted using the Ventana PATHWAY HER2 antibody (rabbit monoclonal, clone 4B5; Ventana, Tucson, AZ, USA). The Food and Drug Administration-approved Ventana PATHWAY is scored from 0 to 3+. Staining in <10% of tumor cells is scored as showing no overexpression (0 or 1+). Strong, complete, circumferential membrane staining in >30% of tumor cells is considered overexpression and is designated as strong positive (3+). Strong circumferential membrane staining in <30% of tumor cells, or circumferential but less than strong staining in any proportion of tumor cells, is designated as equivocal (2+). All immunohistochemical assays were conducted in parallel with known positive and negative controls. The slides were observed using a Nikon Eclipse 80i microscope (Nikon Instruments Inc., Melville, NY, USA). Pictures were taken using a Nikon Digital Camera DXM1200F and images were obtained using Nikon ACT-1 software.

### Array CGH and *PIK3CA* mutation analysis

Genomic DNA was extracted from patient or xenograft tumor samples using DNeasy Blood & Tissue Kit (Qiagen, Valencia, CA, USA). Array CGH analyses were performed at SciGene (Sunnyvale, CA, USA) using Human Genome CGH Microarray 4x44K (Agilent Technologies, Santa Clara, CA, USA), and processed on SciGene's robotic aCGH workstations (ArrayPrep® Target Preparation System, Mai Tai® Hybridization System, and Little Dipper® Processor, SciGene, Sunnyvale, CA, USA).

Mutations were detected by sequencing PCR products derived from amplification primers in the introns flanking *PIK3CA* exons 1, 2, 3, 5, 6, 7, 9, 18 and 20 using Ampli Taq Gold DNA polymerase (Applied Biosystems-Life Technologies, Carlsbad, CA, USA). The primer sets used in these reactions are listed in Table S1 in Additional file 1. Exons that had sequence homology with a known *PIK3CA* pseudogene were not sequenced; however, the sequenced exons included all common mutation hotspots. The reaction was run using a touchdown PCR protocol where the annealing temperature was started at 63°C and decreased for 0.5°C per cycle for 12 cycles. Then the reaction was continued for another 25 cycles at 94°C, 30 sec; 58°C, 30 sec; and 72°C, 30 sec per cycle. PCR products were checked by 2% agarose gel against a GeneRuler 50 bp DNA Ladder (Frementas, Glen Burnie, MD, USA) and sequenced by BigDye Terminator v3.0 Cycle Sequencing Kits (Applied Biosystems-Life Technologies, Carlsbad, CA, USA). The sequencing results were analyzed with Sequencher 4.8 software (Gene Codes Corporation, Ann Arbor, MI, USA).

### Microarray and TNBC subtype analysis

Frozen tumor tissues from xenografts were cut into small pieces on dry ice. RNA was extracted using RNeasy Plus Mini Kit (Qiagen) following the manufacturer’s instructions. The quantity and purity of the RNA sample was measured using the Agilent 2100 bioanalyzer (Agilent Technologies). RNA samples were submitted to Stanford Protein and Nucleic Acid core facility for microarray analysis using Affymetrix GeneChip Human Genome U133 Plus 2.0 arrays. All xenograft microarray datasets are posted on GEO under accession number GSE47079 [47].

TNBC subtyping was done following the Pietenpol group’s methods [7,8]. The microarray data of the patient derived xenograft tumors were robust multi-array average (RMA) normalized and log transformed. For genes containing multiple probes, the probe with the largest interquartile range across the samples was chosen to represent the gene. The processed samples were uploaded to the website [48], where the samples were subjected to an ER-filter scrutiny and then assigned TNBC subtypes.

### Generation of an *in silico* rapamycin response signature

A rapamycin response signature was generated as described previously [49] using Connectivity Map Build 02 gene expression data of 5 rapamycin treated and 18 control MCF7 cell samples [50]. The Connectivity Map studies highlight the ability to use drug treatment on cell lines to identify a set of genes that reflect response to a drug; therefore, independent of the cell type profiled, a “signature” of drug response can be identified from the data that reflects the response to drug treatment [50]. Multiple studies by other groups and our own have shown that such drug response signatures do also function as drug sensitivity/resistance signatures, with sensitive samples having gene expression patterns more like untreated cells and resistant samples having gene expression patterns more like treated cells [49-52]. Specifically, tumors with dysregulation of genes that are modulated by treatment of rapamycin will be predicted as “sensitive” or “resistant” based on their correlation to gene dysregulation from rapamycin treatment in the Connectivity Map data. This approach, which uses expression data from cell lines before and after treatment with a drug, has the advantage over using expression data from cell lines classified as ‘resistant’ or ‘sensitive’ because cell line data have confounding factors, such as subtype that can affect prediction models. By using prediction models that include genes specific to a particular drug’s response, we are not limited by these confounding factors. Thus, treated cell lines of one subtype (for example, luminal MCF7 cells) may be used to predict drug response in samples of different subtypes (for example, basal-like or HER2-overexpressing cancer cells).

To generate the signature, Mas5 normalized gene expression data were quantile-normalized and log2 transformed and used as the training set. A Bayesian binary regression algorithm was then used on the training set to generate the signature. It was further optimized and internally validated in leave-one-out cross-validation (LOOCV) analysis, which tests each individual sample’s classification by leaving it out of the model and predicting if it is treated or untreated [49,51].

### Validation and application of the rapamycin response signature

For signature external validation, CEL files were downloaded from GEO GSE18571, which contains gene expression data for both *in vitro* and *in vivo* rapamycin treatment samples on TNBC cell line MDA-MB-468 [26], and from Connectivity Map batches 2, 35, 44, 56, 63, 70, 626, 757 and 767, and analyzed as described by Cohen *et al*. [49]. We then confirmed the ability of the rapamycin response signature to predict sensitivity to rapamycin *in vitro* by comparing the EC50 (see below) for a diverse panel of cell lines to the predicted sensitivity, that is, similarity to untreated cells, based on gene expression.

As previously described [49], 18 breast cancer cell lines were obtained from ATCC (HCC38, HCC1806, HCC1428, HCC1143, BT483, BT549, BT474, MDA-MB-361, MDA-MB-157, MDA-MB-435S (now considered to be of melanoma origin), MDA-MB-231, MDA-MB-453, SKBR3, ZR75, CAMA I, MCF7, Hs578t, T47D) and used for dose-response assays. Cells were seeded in 384-well plates (Nunc, Rochester, NY, USA) in MEBM media (Lonza, Walkersville, MD, USA) containing 5% fetal bovine serum (Gibco, Grand Island, NY, USA), at a density to yield 80% confluency in control-treated wells at 96 h post-treatment (as determined by growth curves). After 24 h, rapamycin was added at 10 doses of 0, 0.1 pM, 0.3 pM, 1 pM, 3 pM, 10 pM, 30 pM, 100 pM, 300 pM and 1 nM. A BIOMEK 3000 (Beckman Coulter, Indianapolis, IN, USA) robot was used to seed the cells and dispense the drug. After 96 h, CellTiter-Blue Reagent (Promega, Madison, WI, USA) was added to test cell viability. After 2 h of incubation at 37°C, the fluorescence was recorded (560(20)_Ex_/590(10)_Em_) using a Victor3V 1420 Multilabel Counter (Perkin-Elmer, Waltham, MA, USA) plate reader. After subtracting background fluorescence, EC50 was calculated using GraphPad Prism v5 (GraphPad Software, La Jolla, CA, USA) to fit a constrained sigmoidal dose-response curve. Predicted sensitivity for these cell lines was computed using gene expression data from Cohen *et al*. [49] and applying the Bayesian binary regression model. Detailed methods for running the regression model are given in [53]. Predicted sensitivity was compared to actual EC50 using linear regression.

To examine the relationship between intrinsic subtype and rapamycin sensitivity, 1,401 breast cancer samples from eight microarray studies were then analyzed (Table S2 in Additional file 2, duplicate samples were removed from GEO datasets GSE6532, GSE7390 and GSE3494). Intrinsic breast cancer subtypes were assigned as previously described [49,54,55]. Rapamycin sensitivity of the patient breast cancer sample was calculated as described by Cohen *et al*. [49]. A detailed method, the input files, output files and the logistic regression program used in this study are available in [53].

### *In vivo* TNBC xenograft drug treatment experiments

Rapamycin and CCI-779 (LC Laboratories, Woburn, MA, USA) were stored as 50 mg/ml solutions in 100% ethanol at -80°C. The stored solutions were diluted in PBS containing 4% ethanol, 5% polyethylene glycol 400 and 5% Tween 80 for treatment. When tumor xenografts grew to an average between 50 to 100 mm^3^ in tumor volume, mice were stratified and randomized by tumor volume into treatment groups of 5 to 10 mice each. The treatment groups included: 1) rapamycin (sirolimus) group, receiving intraperitoneal (IP) administration of 7.5 mg/kg rapamycin every other day for up to six weeks; 2) CCI-779 (temsirolimus) group, receiving IP administration of 7.5 mg/kg of CCI-779 every other day for up to four weeks; 3) control group, receiving IP administration of control vehicle for up to four weeks; and, in some sets of experiments, 4) a doxorubicin group, receiving IP administration of 2 mg/kg doxorubicin (Sigma-Aldrich, St. Louis, MO, USA) diluted in PBS once every week for three weeks. Tumors were measured twice a week with a caliper in two dimensions. Tumor volume was calculated by the following formula: tumor volume = (*l* x *w*^2^)/2, where *l* is the longest diameter of the tumor, *w* is the shortest diameter of the tumor. Mean tumor volumes were calculated, and growth curves were established as a function of time. The error bars indicated the value of the standard error of the mean. The Student’s *t*-test was used for statistical analysis. We considered *P* <0.05 as statistically significant.

### Protein extraction and Western blot analysis

For protein extraction, frozen xenograft tumors were gently thawed and washed in ice-cold PBS. They were then homogenized using a glass homogenizer and lysed in radio-immunoprecipitation assay buffer containing protease and complete phosphatase inhibitors (Roche, West Sussex, UK). After quantification using Pierce’s BCA protein assay (Thermo Scientific Pierce, Leicestershire, UK), 5 to 10 μg total proteins were run on 4 to 12% SDS-PAGE gels (NuPAGE Bis-Tris Gels, Invitrogen-Life Technologies, Paisley, UK), then immunoblotted with the antibodies for PTEN, mTOR, p-mTOR (Ser 2448), 4EBP1, p-4EBP1 (Ser 65), S6K1, p-S6K1 (Thr 389), eIF4E and p-eIF4E (Ser 209) at 1:1,000 dilutions. Secondary horseradish peroxidase (HRP)-labeled antibodies were used at 1:5,000 dilutions. Tubulin was used as a loading control. All antibodies were obtained from Cell Signaling Technology (Hitchin, Hertfordshire, UK). Membranes were visualized by the ECL developer system (GE Healthcare Life Sciences, Piscataway, NJ, USA). Protein expression was quantified by analyzing a representative autoradiograph with Image Image J software (public domain software developed at the Research Services Branch, National Institute of Mental Health, Bethesda, MD, USA) [56].

## Results

### Patient-derived orthotopic xenograft models of TNBC

We developed a panel of seven TNBC xenograft tumors from six patients, generated from six primary tumors (SUTI097, SUTI103, SUTI110, SUTI151, SUTI319, SUTI368) and one soft tissue metastasis in the quadriceps muscle of the leg (SUTI151M). In general, these xenografts represented aggressive TNBC. Most patients from whom the xenografts were derived had poor disease-free survival despite treatment with multiple standard therapies, with four of five patients who had follow-up greater than one year showing metastatic recurrence or death from disease (Table S3 in Additional file 3).

**Figure 1:**
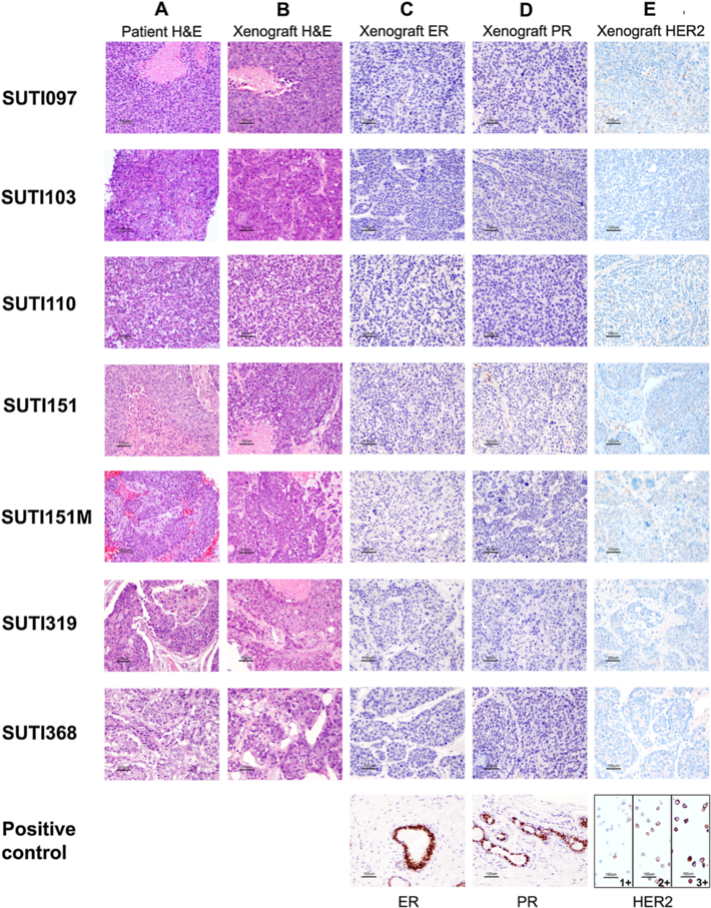
**Histology of patient TNBC samples and corresponding patient-derived orthotopic xenografts. A**. H&E staining of patient tumors; **B**. H&E staining of the corresponding xenograft tumors; **C**. ER staining of xenograft tumors; **D**. PR staining of xenograft tumors; and **E**. HER2 staining of xenograft tumors. Pictures were taken with 200× magnification. The scale bar is 100 μm in length. ER, estrogen receptor; HER2, human epidermal growth factor receptor 2; PR, progesterone receptor.

We compared the xenograft tumors to the corresponding patient tumors by histology, clinical biomarker expression, genome-wide array CGH, and *PIK3CA* sequencing. Based on hematoxylin and eosin (H&E) staining (Figure 1A, B), the original TNBC tumors exhibited a variety of histologies that were conserved in the corresponding xenografts. Specifically, there were similarities in cancer cell morphology, mitotic index, stromal abundance and percent necrosis. As expected, all xenografts were confirmed to be triple-negative by ER, PR and HER2 staining using the same clinical laboratory protocols as were performed on the patient samples (Figure 1C-E).

**Figure 2:**
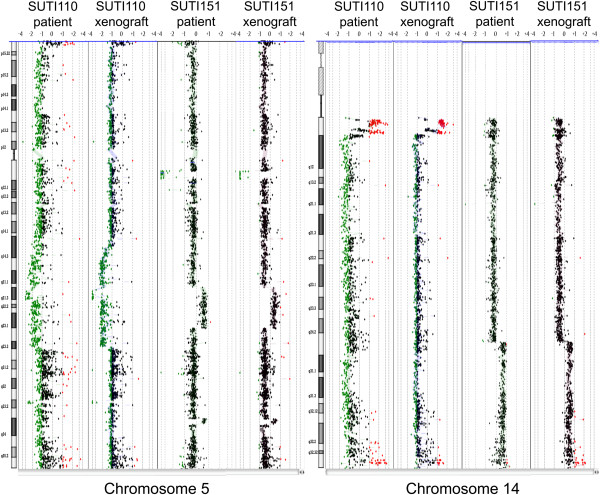
**Array CGH profiling.** Data for SUTI110 and SUTI151 show matching variations on chromosomes 5 and 14 for patient tumors and corresponding xenografts. Green represents loss and red represents gain for each probe aligned along the chromosome. Other chromosome profiles are provided in Figure S1 in Additional file 4. CGH, comparative genomic hybridization.

To compare global genomic profiles between patient tumors and their corresponding xenografts, we performed array comparative genomic hybridization (aCGH) on two tumors, SUTI110 and SUTI151. The xenograft tumors faithfully maintained the genomic DNA alterations observed in the corresponding patient tumors (Figure 2 and Figure S1 in Additional file 4). Interestingly, a previously unreported 5q11-12 deletion was observed in both patient tumor SUTI151 and its corresponding xenograft (Figure 2), and was also maintained in the xenograft of the soft tissue metastasis, SUTI151M, that developed months later (data not shown).

**Figure 3:**
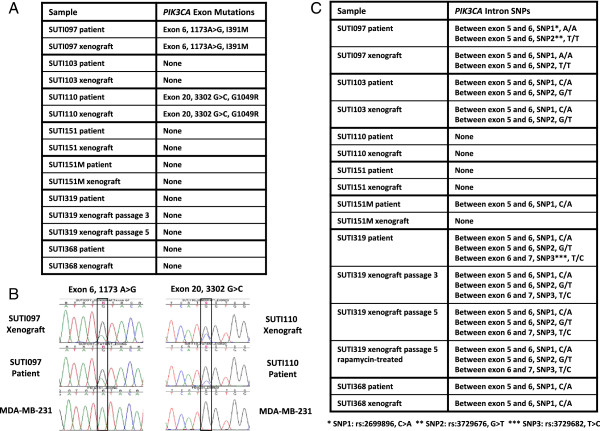
***PIK3CA *sequence variations. A**. This is a table of *PIK3CA* exon mutations in patient and corresponding xenograft samples. The exon number, mRNA position and allele change, and protein position and residue change for each mutation are indicated. **B**. A sequencing image of patient and corresponding xenograft tumors of the SUTI097 and SUTI110 at the mutated sites. MDA-MB-231 cell line is shown as a normal control. **C**. Table of *PIK3CA* intron SNPs that flank sequenced exons in patient and corresponding xenograft samples. Note the conservation of sequence variations between primary tumors and their xenografts, and also between different xenograft passages, before and after rapamycin treatment. SNPs, single nucleotide polymorphisms.

*PIK3CA* sequencing showed that two of the seven (29%) patient primary and metastatic tumors and xenografts contained missense mutations (Figure 3A, B). SUTI097 patient and xenograft samples contained an exon 6 mutation (1173 A > G, I391M); SUTI110 patient and xenograft samples contained an exon 20 mutation (3302 G > C, G1049R). We did not observe any exon 9 mutations, which are common mutation sites in ER positive tumors.

We also detected multiple SNPs in introns flanking the sequenced *PIK3CA* exons, and these sequence variations were also maintained between all primary tumors and their corresponding xenografts (SUTI097, SUTI103, SUTI319, SUTI368, Figure 3C). In the one soft tissue metastasis, however, the metastatic tumor showed an additional SNP not present in the primary tumor or in the xenografts generated from the patient’s primary or metastatic tumors (SUTI151 and SUTI151M). When sequence variations were analyzed between multiple xenograft tumor passages for primary tumor SUTI319, passage 3 of its xenograft tumor and passage 5 of its xenograft tumor, both before and after rapamycin treatment, there was conservation of three SNPs identified in introns between exons 5 and 6, and between exons 6 and 7 (Figure 3C). In summary, our xenografts recapitulated the histology, biomarker status, genomic profile and *PIK3CA* sequence of corresponding primary patient tumors.

### The xenograft models represent multiple TNBC subtypes

Human TNBCs have been shown to be heterogeneous, comprised of at least six stable subtypes and a possible seventh unstable subtype [7]. To determine the subtypes of our panel of TNBC xenografts, we performed microarray analyses on the xenograft tumors and classified them according to the method developed by Pietenpol’s group [7,8]. As shown in Table 1, of our panel of seven TNBC xenograft tumors, five xenografts subclassified into four of the six stable subtypes; two were classified with Pietenpol’s “unstable” group. In particular, SUTI097 belonged to the immunomodulatory (IM) subtype; SUTI103 and SUTI110 were classified as basal-like 1 (BL1); SUTI151 was classified as basal-like 2 (BL2); SUTI151M, the soft tissue metastasis of SUTI151, was identified as mesenchymal (M) subtype; and SUTI319 and SUTI368 clustered with the “unstable” group of TNBCs. We find it anecdotally fitting that the metastasis of a BL2 subtype subclassified as M subtype, which is associated with increased expression of genes involved in cell motility, cellular differentiation, growth pathways and TGF-β signaling [7]. Our xenograft panel thus represents a majority of TNBC subtypes, making it suitable for pre-clinical drug testing.

**Table 1:** Classification of xenograft tumors based on TNBC subtypes

**Xenograft ID**	**TNBC subtype**
**SUTI097**	immunomodulatory (IM)
**SUTI103**	basal-like 1 (BL1)
**SUTI110**	basal-like 1 (BL1)
**SUTI151**	basal-like 2 (BL2)
**SUTI151M**	mesenchymal (M)
**SUTI319**	unstable (UNS)
**SUTI368**	unstable (UNS)

TNBC, Triple-negative breast cancer.

### Rapamycin response signature and its validation

**Figure 4:**
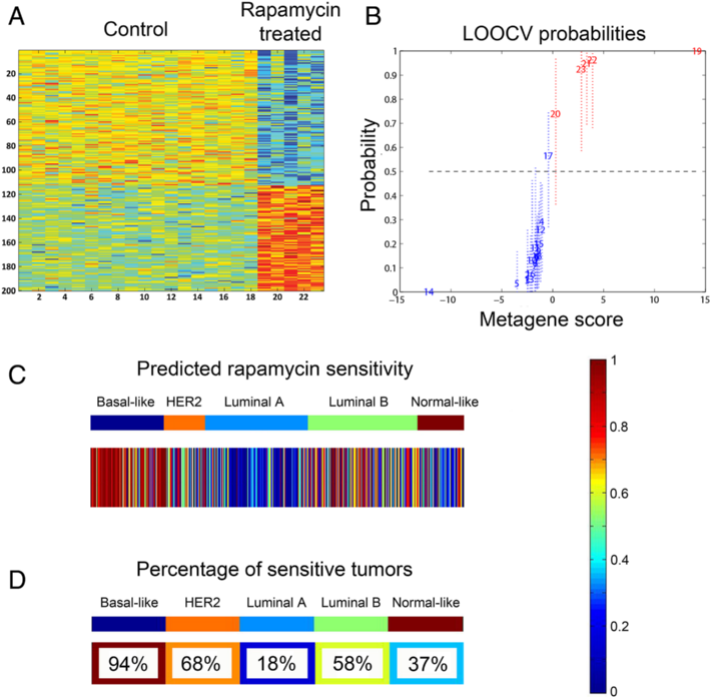
**Rapamycin response signature. A**. Heatmap of rapamycin response signature gene expression of training set samples with 18 control samples on the left and 5 rapamycin treated samples on the right. Each row is a probe set in the signature. Red indicates up-regulation and blue indicates down-regulation of the gene. **B**. LOOCV from the Connectivity Map training set samples. On the y-axis, 0 = predicted as untreated; 1 = predicted as treated. Control samples are in blue, and rapamycin treated samples in red. Note that only one control sample was misclassified. **C**. Heatmap of predicted rapamycin response of 1,401 human breast tumors with a color scaled from red to blue indicating a high to low predicted sensitivity. Each column represents an individual tumor sample, grouped by intrinsic subtypes. **D**. The percent of samples with predicted rapamycin sensitivity of >0.5 for each intrinsic subtype. The background color represents the overall sensitivity of each subtype at the same scale used in 1C. LOOCV, leave-one-out cross-validation.

To explore how commonly rapamycin sensitivity is expected to occur among diverse breast cancers, including TNBC, we developed a rapamycin response signature that would predict sensitivity or resistance based on a cancer’s gene expression. This builds on our previous work with gene expression-based signatures, which were derived using the same approach, and which showed that drug response signatures predict whether a tumor or cell line will be sensitive or resistant to a drug [49,51,52]. The rapamycin response signature contained 200 probe sets and represented 175 unique genes (See Table S4 in Additional file 5). Figure 4A shows the heatmap view of the expression pattern of the probe sets from the signature in the training set samples from the Connectivity Map database [50] displaying expression changes in response to rapamycin treatment. When analyzed by leave-one-out cross validation, a method that leaves a single sample out to predict whether it will be classified as treated (probability >0.5 on the y-axis) or untreated (probability <0.5 on the y-axis), 22 of 23 training set samples showed the expected prediction, including all five treated cell line samples (Figure 4B), indicating high consistency and robustness of our signature.

To validate our rapamycin response signature in TNBC, we next tested its accuracy on an independent external dataset containing both *in vitro* and *in vivo* rapamycin treatment samples that were generated from the TNBC cell line MDA-MB-468 [26]. For both the *in vitro* MDA-MB-468 cells and the 22-day xenografts, the treated samples were all correctly predicted to be more like the treated samples in the signature training set, and hence more resistant to further rapamycin treatment, than the untreated MDA-MB-468 cells (*P* = 0.0017) and xenografts (*P* = 0.06) (Figure S2A in Additional file 6). These data confirm that the rapamycin response signature can distinguish TNBC cells that have been treated with rapamycin from untreated TNBC cells.

We also tested the rapamycin response signature on other samples in the Connectivity Map that had available treatment data on rapamycin as well as other drugs. As shown in Figure S2B in Additional file 6, only samples treated with rapamycin or PI3K inhibitors showed the expected rapamycin response signature post-treatment pattern whereas the samples treated with random drugs did not (*P* <0.0001).

Finally, to show that the response signature does predict sensitivity and resistance to rapamycin, we compared predictions of the rapamycin response signature in a panel of 18 breast cancer cell lines to the actual EC50 obtained when these cells were treated with rapamycin. As shown in Figure S3 in Additional file 6, we found a significant correlation between signature prediction and *in vitro* drug sensitivity as measured by EC50 (r = -0.3; *P* = 0.02). Therefore, the response signature was confirmed to also be a predictor of sensitivity to rapamycin.

### Rapamycin sensitivity in intrinsic breast cancer subtypes

We then used the rapamycin response signature to perform a supervised analysis of eight published gene expression datasets including 1,401 breast cancer samples from patients that were classified into the five intrinsic molecular subtypes [54]. As shown in Figure 4C, D, 94% of BLBCs, 68% of HER2-overexpressing tumors, 18% of luminal A tumors, 58% of luminal B tumors and 37% of normal-like tumors were predicted to be sensitive to rapamycin. The predicted rapamycin sensitivity differed between subtypes, with the more aggressive subtypes - such as basal-like, HER2-overexpressing and luminal B - having much more frequently predicted sensitivities. Among them, BLBC had the highest frequency of rapamycin-sensitive tumors. Signatures for other PI3K pathway inhibitors showed similar patterns of predicted drug response among the different subtypes (data not shown).

### Rapamycin and CCI-779 significantly inhibit tumor growth in our TNBC xenograft models

**Figure 5:**
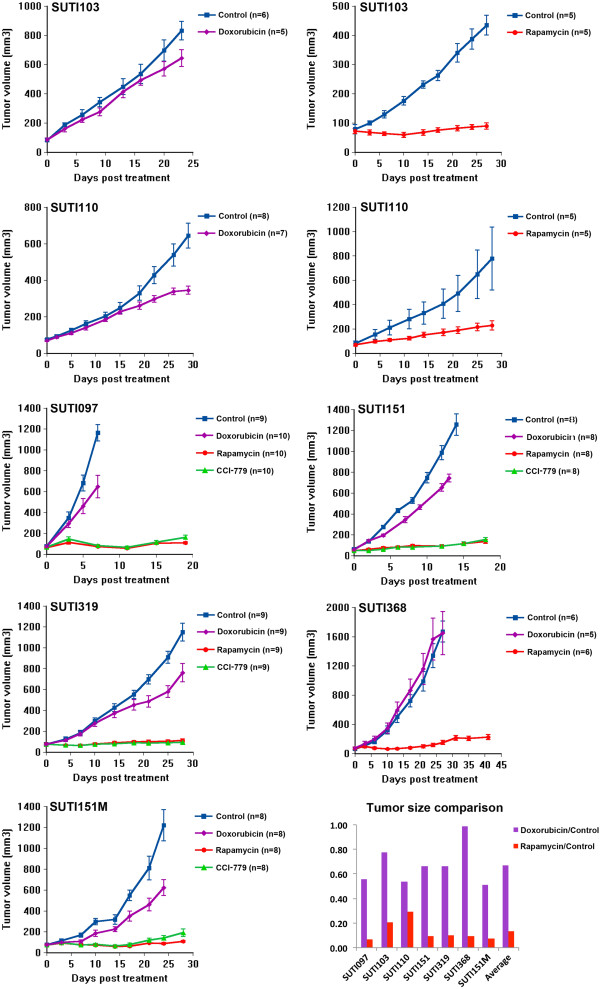
***In vivo *growth curves of seven patient-derived orthotopic xenografts of TNBC.** Treatment with vehicle control in blue; doxorubicin in purple; rapamycin in red; CCI-779 in green. Tumor volumes in mm^3^. Each data point represents the mean tumor volume of each treatment group. Error bars represent standard error of the mean. CCI-779, temsirolimus; TNBC, triple-negative breast cancer.

We next used our panel of seven TNBC xenografts to evaluate rapamycin sensitivity *in vivo*, measuring growth inhibition of two mTOR inhibitors, rapamycin (sirolimus) and/or its analog, CCI-779 (temsirolimus) and doxorubicin, a drug widely used to treat TNBC. Compared to untreated tumors, growth rates of doxorubicin-treated tumors in all seven orthotopic xenograft models showed only a minimum to partial response, with growth inhibition ranging from 2 to 52% (Figure 5 and Table S3 in Additional file 3). In sharp contrast, the overall efficacy of rapamycin and CCI-779 was significantly higher than doxorubicin for the treatment of these TNBC xenografts (*P* = 0.0003, Figure 5). On average, rapamycin inhibited tumor growth by 94% (range 77 to 99%), whereas the average inhibition by doxorubicin was only 36% (Figure 5, and Table S3 in Additional file 3). This supports the hypothesis that TNBCs are highly sensitive to rapamycin. For the four xenografts treated with both mTOR inhibitors, drug efficacy was similar. However, none of the tumors disappeared completely, with most maintaining a small volume of residual tumor, suggesting that additional drugs may be necessary in combination with mTOR inhibitors to totally ablate residual disease.

### mTOR pathway activation in patient-derived TNBC xenografts

**Figure 6:**
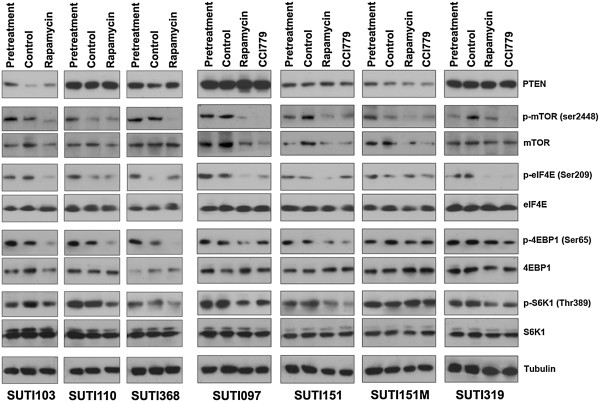
**Protein expression and phosphorylation of PTEN, mTOR, S6K1, 4EBP1, and eIF4E in xenografts.** Western blot images were cropped at the molecular weight of each of the target proteins. Pretreatment samples were collected prior to initiation of treatment; control samples (vehicle control), rapamycin-treated and CCI-779-treated samples were collected at the end of the treatment period. Tubulin was used as a loading control. See also Table S5 in Additional file 7. CCI-779, temsirolimus.

To identify pathway activation, we performed Western blot analyses on PTEN and other mTOR pathway proteins. As shown in Figure 6, the protein levels of PTEN vary among the xenografts. SUTI097, SUTI110, SUTI319 and SUTI368 have relatively higher PTEN protein levels than xenografts SUTI103, SUTI151, SUTI151M, although all samples exhibited *in vivo* sensitivity to mTOR inhibitors (Figure 5). The PTEN levels generally remained consistent pre- and post-treatment and its expression did not appear to be associated with any particular TNBC subtype.

Phosphorylated mTOR and its downstream proteins - 4EBP1, S6K1 and eIF4E - were detected in all xenograft samples (Figure 6), demonstrating baseline mTOR pathway activation. Treatment with one or both mTOR inhibitors decreased phosphorylation of mTOR and, to varying extents, its downstream proteins. The exception was metastatic tumor xenograft SUTI151M (Figure 6), although both treatments still inhibited its growth by over 90%, raising the possibility of other potential modes of action of mTOR inhibitors. For all primary tumor xenografts, the overall phosphorylation of mTOR, S6K1, 4EBP1 and eIF4E proteins was decreased by 53%, 33%, 62% and 64%, respectively, in rapamycin-treated tumors compared with tumors in the pretreatment and control groups (Table S5 in Additional file 7), supporting decreased mTOR pathway activity after treatment.

## Discussion

Developing more effective therapies would be of significant benefit to patients with TNBC. We describe here multiple patient-derived orthotopic xenograft models that molecularly mimic patients’ original tumors and represent diverse TNBC subtypes. We use these to demonstrate the promising potency of mTOR inhibitors as suggested by *in silico* testing of a rapamycin response signature generated by our group.

We demonstrated that our models closely recapitulated original patient tumors morphologically, by molecular biomarkers, global copy number variation and *PIK3CA* sequencing. Such patient-derived models have also been demonstrated by others to faithfully maintain histology [57-63], gene expression patterns [60-63] and genomic features [57,58,61,63,64] in diverse human breast cancers, including triple-negative, ER positive and HER2-overexpressing tumors. These models have also been shown to be effective for preclinical therapeutic studies [45,46,57,58,62,63,65,66].

By sequencing, we observed *PIK3CA* mutations in two of our seven (29%) patient and xenograft pairs of TNBC tumors, with mutations in exon 6 (I391M, n = 1), and exon 20 (G1049R, n = 1). We also noted complete conservation of multiple SNPs in the flanking introns adjacent to the sequenced exons for the primary tumors, even on subsequent xenograft passages. The soft tissue metastasis of one of the primary tumors contained an intron SNP, which was not observed in the primary tumor or in the xenografts of the primary or metastatic tumor. The reason for this is unclear but may reflect lack of depth of our sequencing or increased heterogeneity in metastases.

Whole exome sequencing of 93 basal-like breast cancers by the Cancer Genome Atlas Network [34] identified *PIK3CA* mutations in 9 (10%). These were present in exon 1 (R88Q, n = 1; R108H, n = 1), exon 4 (N345K, n = 1), exon 9 (E542K, n = 1), exon 12 (F614I, n = 1) and, most commonly, exon 20 (H1047R, n = 4), none of which were detected in our panel. Another sequencing series has reported a 10% *PIK3CA* mutation rate in 65 TNBCs [35], with one mutation in exon 9 (E545K) and most in exon 20 (H1047R). For technical reasons, whole exome sequencing may not always identify mutations when the mutant cells make up less than 10% of the sample or because of lack of adequate sequencing coverage or depth. Thus, when a mass spectroscopy approach evaluated SNPs for 23 known site-specific mutations in *PIK3CA*, 8% of 240 TNBCs revealed mutations located in exon 4 (N345K), exon 7 (E418K), exon 9 (E545K, E542K, P539R) and exon 20 (H1047R, H1047L, H1047Y, G1049R) [67]. In sum and including our tumors, *PIK3CA* mutations in TNBC have now been identified in exons 1, 4, 6, 7, 9, 12 and 20. We also note that despite the known genomic instability of TNBCs [68], we observed that all *PIK3CA* sequence variations persisted between patient primary tumors and xenograft models, and between xenograft models assayed during different sequential passages.

Our seven patient-derived xenograft models spanned different TNBC subtypes as described by Pietenpol’s group [7,8], who analyzed gene expression profiles of 587 TNBCs from 21 datasets to determine different TNBC subtypes. They identified six stable subtypes and an unstable subtype (UNS). The stable subtypes included two basal-like (BL1 and BL2), an immunomodulatory (IM), a mesenchymal (M), a mesenchymal stem-like (MSL), and a luminal androgen receptor (LAR). Using their analytic tools, we found that five of our seven TNBC xenografts represented four stable subtypes (BL1, BL2, M and IM), and two were in the UNS group, confirming our panel’s subtype diversity. Chang’s group recently analyzed 15 patient-derived TNBC xenografts and found that 12 spanned three subtypes (BL1, n = 8; M, n = 3; BL2/IM, n = 1) with three xenografts unclassified [69]. MSL and LAR subtypes were not identified in our or Chang’s series of patient-derived xenograft models.

Interestingly, we found that a xenograft generated from a primary tumor (SUTI151) was classified as basal-like 2 (BL2), whereas the xenograft generated from its soft tissue metastasis (SUTI151M) was classified as mesenchymal (M). The BL2 subtype expresses genes involved in growth factor signaling, glycolysis and gluconeogenesis, whereas the M subtype is enriched for genes involved in cell motility, extracellular matrix receptor interaction and cell differentiation pathways, including the Wnt pathway, anaplastic lymphoma kinase (ALK) pathway and TGF-β signaling [7]. This adds support to the idea that distant metastases acquire different signaling programs than the primary tumor.

Here, we developed and validated a rapamycin response signature that predicts sensitivity and resistance to rapamycin. The signature predicted that the majority of BLBCs should be sensitive to rapamycin, suggesting activation of the mTOR pathway in this subtype. This is consistent with data from the Cancer Genome Atlas Network group [34]. They analyzed PI3K pathway activation in 390 human breast tumors across five intrinsic subtypes using mRNA expression signatures from different sources. Signatures from both Saal *et al*. (PTEN loss in human breast tumors) and Connectivity Map (PI3K/mTOR inhibitor treatment *in vitro*) showed similar patterns: the basal-like subtype had the highest PI3K pathway activity and luminal A had the lowest pathway activity [32,34]. These results agree with our rapamycin response signature predictions. In addition, they show that BLBCs have the highest expression levels of PI3K/AKT pathway genes, as well as a high *PIK3CA* gene amplification rate (49%) [34]. Also consistent is that protein levels of the mTOR pathway suppressors, PTEN and INPP4B, are relatively low in BLBC or TNBC patient tumors compared with other breast cancer subtypes [14,32,34,36]; and mTOR pathway-related proteins, especially AKT and 4EBP1, show high phosphorylation levels in BLBCs [33,34]. Moreover, Moestue *et al*. recently demonstrated that BEZ235, a dual PI3K/mTOR inhibitor, had potent *in vivo* efficacy in a patient-derived BLBC xenograft model, but not in a luminal model [70], also supporting our findings.

Clinically, breast cancers are more commonly classified by their biomarkers (ER, PR and HER2) rather than by microarray analysis. As described above, most TNBCs (about 70 to 80%) are basal-like subtypes by gene expression analysis. It is thus reasonable to expect high rapamycin sensitivity among TNBCs according to our prediction model. This was confirmed by a remarkable 77 to 99% growth inhibition of either drug (mean 94%), whereas the average inhibition by doxorubicin was only 36%.

Supporting our growth inhibition findings, we showed that the mTOR pathway was activated in all our TNBC patient-derived xenografts, as indicated by the phosphorylation of mTOR and downstream proteins 4EBP1 and S6K1. This is consistent with observations in human TNBCs [33,34]. After treatment of the xenografts generated from primary tumors, overall decreased phosphorylation of these proteins suggested decreased mTOR pathway activity, which may have contributed to observed tumor growth inhibition. We observed that mTOR inhibitor treatment exerted a greater decrease in 4EBP1 phosphorylation (62%) than in S6K1 phosphorylation (33%), although individual tumor responses varied.

In our study, mTOR inhibitors showed a cytostatic effect on tumor growth (growth inhibition) but did not reduce original tumor volume over time. To obtain tumor shrinkage or complete ablation, it is likely that additional drugs need to be added. Supporting this is a negative Phase II single drug study of another mTOR inhibitor, everolimus, which did not show partial or complete responses in any of five ER negative/HER2 negative patients with metastatic breast cancer [71]. In contrast, a recent phase II clinical trial evaluating temsirolimus and carboplatin achieved a 36% clinical benefit rate of patients with metastatic triple-negative breast cancer [42]. As well as investigating the addition of mTOR inhibitors to current therapies, new drug combinations are also under study, such as mTOR catalytic inhibitors, dual kinase inhibitors of mTOR and PI3K, and combined targeting of the selective allosteric pan-AKT inhibitor MK-2206 with mTOR inhibition [70,72-76]. We are optimistic that mTOR inhibitors will broadly affect the treatment of breast cancer, especially TNBCs.

## Conclusions

In summary, we generated seven patient-derived orthotopic xenograft models of TNBC that matched original patient primary and metastatic tumors by histology, biomarkers, genomic features and *PIK3CA* sequencing. These models spanned at least four of six TNBC subtypes. We developed a rapamycin response signature that predicted sensitivity in BLBCs. Testing two mTOR inhibitors in our TNBC xenograft models, we confirmed *in vivo* growth inhibition in all. Our data suggest that mTOR pathway inhibition warrants further preclinical and clinical investigation in TNBC in conjunction with other drugs.

## Abbreviations

4EBP1: eukaryotic translation initiation factor 4E binding protein 1; aCGH: array comparative genomic hybridization; AKT: protein kinase B; BL1: basal-like 1; BL2: basal-like 2; BLBC: basal-like breast cancer; CCI-779: temsirolimus; DMSO: dimethyl sulfoxide; EGFR: epidermal growth factor receptor; ER: estrogen receptor; FBS: fetal bovine serum; H&E: hematoxylin and eosin; HER2: human epidermal growth factor receptor 2; IM: immunomodulatory; INPP4B: inositol polyphosphate-4-phosphatase, type II; IP: intraperitoneal; LAR: luminal androgen receptor; LKB1: serine-threonine kinase 1; LOOCV: leave-one-out cross-validation; M: mesenchymal; MSL: mesenchymal stem-like; mTOR: mammalian target of rapamycin; PBS: phosphate-buffered saline; PI3K: phosphatidylinositide 3-kinase; PIK3CA: phosphatidylinositol-4,5-bisphosphate 3-kinase, catalytic subunit alpha; PR: progesterone receptor; PTEN: phosphatase and tensin homologue; S6K1: p70 ribosomal S6 kinase 1; SNP: single nucleotide polymorphism; TGF-β: transforming growth factor-beta; TNBC: triple-negative breast cancer; TSC: tuberous sclerosis complex; UNS: unstable.

## Competing interests

The authors declare that they have no competing interests.

## Authors’ contributions

HZ, SSJ, AHB, ALC and GVG conceived the study and MLT and SHD helped with its design. ILW, FMD and SSJ provided patient tumor tissue and clinical data, and ILW, GVG and SSJ performed clinical data analyses. HZ generated tumor xenografts and prepared samples for microarray analysis. TAL analyzed all pathology data. GD and MH performed and analyzed the array CGH studies, with additional interpretation and compilation by DOF. HZ and SK performed and analyzed DNA sequencing. MAC performed tumor subtyping using microarray data. ALC and AHB developed and validated the drug response signature, applied the signature to breast tumor datasets, and analyzed results. HZ and CMP performed *in vivo* drug testing and data acquisition. HZ, SSJ and GVG interpreted drug response in xenografts. SV and BL performed and analyzed Western blots, with additional interpretation by ACM. HZ, SSJ, ALC, AHB, GVG, SK, ILW, SV, GD, MAC, TAL, DOF and BL wrote the manuscript, while CMP, MH, MLT, FMD, ACM and SHD provided further input to the manuscript and/or critical revisions. All authors read, commented on and approved the final manuscript.

## Supplementary Material

Additional file 1: Table S1 Primer sets for *PIK3CA* mutational analyses. Click here for file

Additional file 2: Table S2 GEO breast cancer microarray datasets used in Figure 4. Click here for file

Additional file 3: Table S3 Summary of therapeutic responses in patient-derived xenograft models of TNBC and clinical responses to standard chemotherapy. Click here for file

Additional file 4: Figure S1 Array CGH profiles of all chromosomes comparing patient (p) and xenograft (x) for samples SUTI 151 and SUTI 110. Click here for file

Additional file 5: Table S4 Rapamycin response signature probes. Table showing 200 Affymetrix probes making up the rapamycin response signature. The probes are annotated with gene name, gene symbol and weight given to each probe relative to the first principal component in the rapamycin-response signature. Click here for file

Additional file 6: Figure S2 Validations of rapamycin response prediction. **A**. Plots of predicted rapamycin sensitivity of MDA-MB-468 cells based on GEO data set GSE18571. As indicated, MDA-MB-468 was treated with either vehicle control (DMSO) or rapamycin in both cell culture and xenografts. Xenograft tumors were collected after 1 day or 22 days of treatment. **B**. Plots of predicted sensitivity to rapamycin in Connectivity Map samples from nine independent batches. Samples are grouped as untreated controls (Untreated), rapamycin-treated (Rapamycin), PI3K inhibitors-treated (PI3K inhibitors), or treated with drugs other than rapamycin or PI3K inhibitors (Other drugs). The bar showed the mean of the predicted sensitivity with 1 as the highest and 0 the lowest predicted sensitivity to rapamycin. **Figure S3** Correlation of actual sensitivity and predicted sensitivity. Correlation of actual sensitivity to rapamycin treatment (indicated by EC50) and predicted sensitivity by the rapamycin response signature of 18 breast cancer cell lines (scattered dots). A regression line was drawn to show the degree of correlation. Click here for file

Additional file 7: Table S5 Phosphorylation levels of S6K1, 4EBP1, eIF4E and mTOR by immunoblot after rapamycin or CCI-779 treatment. Click here for file
