# Supplementary material for: Patient-derived xenografts of triple-negative breast cancer reproduce molecular features of patient tumors and respond to mTOR inhibition
Source: Breast Cancer Res. 2014 Apr 7;16(2):R36. doi: 10.1186/bcr3640 (PMC4053092; doi:10.1186/bcr3640)
Supplement: Additional file 1: Table S1 — Primer sets for PIK3CA mutational analyses. [file bcr3640-S1.docx]

**Table S1: Primer sets for *PIK3CA* mutational analyses.**

| Amplicon | Forward primer | Reverse primer |
| --- | --- | --- |
| Exon1 | GCCTAATCAAGTCAAACTATGGAAA | TTTTAGAAAGGGACAACAGTTAAGC |
| Exon2 | TCTACAGAGTTCCCTGTTTGC | ATAAGCAGTCCCTGCCTTCA |
| Exon3 | TTGGGCTGATTAAAAAGCAT | CAGATACTCATCCTCAATGTGATT |
| Exon5 | TCTGAACAAAAATTCCGTGGT | AATGGGGTCTTGCTTTGTTG |
| Exon6 | TGGTTGATCTTTGTCTTCGTG | AATTCAATCAGCGGTATAATCAGG |
| Exon7 | TGGGGAAGAAAAGTGTTTTGA | CCAGTAAAATATATGGATCCTTTTCC |
| Exon9 | CTGTGAATCCAGAGGGGA | CAGAGAATCTCCATTTTAGCAC |
| Exon18 | AATGGAAACTTGCACCCTGT | TTGTTTCTAATAGAGCAGCCAGAA |
| Exon20 | TTGCATACATTCGAAAGACC | GGGGATTTTTGTTTTGTTTTG |
