# Supplementary material for: Patient-derived xenografts of triple-negative breast cancer reproduce molecular features of patient tumors and respond to mTOR inhibition
Source: Breast Cancer Res. 2014 Apr 7;16(2):R36. doi: 10.1186/bcr3640 (PMC4053092; doi:10.1186/bcr3640)
Supplement: Additional file 2: Table S2 — GEO breast cancer microarray datasets used in Figure 4. [file bcr3640-S2.docx]

**Table S2: Breast cancer microarray datasets used in Figure 4.**

| **GEO GSE** | **Basal-like** | **Her2+** | **Luminal A** | **Luminal B** | **Normal-like** | **Total** |
| --- | --- | --- | --- | --- | --- | --- |
| GSE2034 | 66 | 36 | 86 | 85 | 13 | **286** |
| GSE2603 | 30 | 13 | 22 | 25 | 9 | **99** |
| GSE3494 | 17 | 16 | 36 | 23 | 22 | **114** |
| GSE1456 | 28 | 12 | 41 | 49 | 29 | **159** |
| GSE5460 | 38 | 20 | 39 | 27 | 3 | **127** |
| GSE6532 | 45 | 35 | 111 | 148 | 73 | **412** |
| GSE7390 | 41 | 13 | 39 | 49 | 12 | **154** |
| GSE7904 | 20 | 8 | 8 | 11 | 3 | **50** |
| **Total** | **285** | **153** | **382** | **417** | **164** | **1401** |
