# Supplementary material for: Patient-derived xenografts of triple-negative breast cancer reproduce molecular features of patient tumors and respond to mTOR inhibition
Source: Breast Cancer Res. 2014 Apr 7;16(2):R36. doi: 10.1186/bcr3640 (PMC4053092; doi:10.1186/bcr3640)
Supplement: Additional file 3: Table S3 — Summary of therapeutic responses in patient-derived xenograft models of TNBC and clinical responses to standard chemotherapy. [file bcr3640-S3.docx]

**Table S3 - Summary of therapeutic responses in patient-derived xenograft models of TNBC**

**and clinical responses to standard chemotherapy**

| Tumor | Xenografts, | Xenografts, | Xenografts, | | Tumor | Positive | Neoadjuvant | Adjuvant and/or | Received | Status | Site of |
| --- | --- | --- | --- | --- | --- | --- | --- | --- | --- | --- | --- |
|  | % inhibition | % inhibition | % inhibition | | size | LNs | chemoRx | Other chemoRx | Doxorubicin/ |  | Metastases |
|  | doxorubicin | rapamycin | CCI-779 | (cm) | |  |  |  | Responded |  |  |
| **TNBC - primary** | | | | | | | | | | | |
| SUTI 097 | 47% (n=10) | 99% (n=10) | 99% (n=10) | | 9.0 w | 2 | Doxorubicin, | Doxil and multiple | Yes/No | DOD | Lung, |
|  |  |  |  | | satellite |  | cyclophosphamide, | other drugs |  |  | mediastinum, |
|  |  |  |  | | lesions |  | taxotere |  |  |  | pericardium |
| SUTI103 | 25% (n=5) | 95% (n=5) | ND | | 4.8 | 0 | N/A | Doxorubicin, | Yes/Yes | NED | N/A |
|  |  |  |  | |  |  |  | cyclophosphamide, |  |  |  |
|  |  |  |  | |  |  |  | paclitaxel |  |  |  |
| SUTI110 | 51% (n=7) | 77% (n=5) | ND | | 7 | 1 | N/A | Refused | No | DOD | Brain, lung, |
|  |  |  |  | |  |  |  |  |  |  | mediastinal |
|  |  |  |  | |  |  |  |  |  |  | lymph nodes, |
|  |  |  |  | |  |  |  |  |  |  | bone |
| SUTI151 | 36% (n=8) | 95% (n=8) | 96% (n=8) | | 2.2, | 1 | Cyclophosphamide, | Capecitabine, | No | DOD | Brain, lung, |
|  |  |  |  | | invading |  | methotrexate, | cyclophosphamide, |  |  | bone, liver, |
|  |  |  |  | | pectoral |  | fluorouracil, paclitaxel, | taxotere, paclitaxel, |  |  | pericardium, |
|  |  |  |  | | muscle |  | gemcitabine, | bevacizumab |  |  | scalp, |
|  |  |  |  | |  |  | carboplatin |  |  |  | quadriceps |
|  |  |  |  | |  |  |  |  |  |  | muscle |
| SUTI319 | 36% (n=9) | 97% (n=9) | 98% (n=9) | | 10 | 13 | Cyclophosphamide, | Carboplatin, | Yes/No | AWD* | Lung, liver, |
|  |  |  |  | |  |  | doxorubicin, | gemcitabine |  |  | abdomen, |
|  |  |  |  | |  |  | fluorouracil, taxotere, |  |  |  | chest wall |
|  |  |  |  | |  |  | carboplatin, |  |  |  |  |
|  |  |  |  | |  |  | gemcitabine |  |  |  |  |
| SUTI368 | 2% (n=5) | 95% (n=6) | ND | | 4.2 | 2 | N/A (octogenerian) | N/A (octogenerian) | No | N/A** | N/A |
| **TNBC - metastatic** | | | | | | | | | | | |
| SUTI151M | 52% (n=8) | 99% (n=8) | 94% (n=8) | | 3.5 | N/A | Cyclophosphamide, | Capecitabine, | No | DOD | Quadriceps |
|  |  |  |  | |  |  | methotrexate, | cyclophosphamide, |  |  | muscle (soft |
|  |  |  |  | |  |  | fluorouracil, paclitaxel, | taxotere, paclitaxel, |  |  | tissue of leg) |
|  |  |  |  | |  |  | gemcitabine, | bevacizumab |  |  |  |
|  |  |  |  | |  |  | carboplatin |  |  |  |  |

*Lost to follow-up after 16 months from first chemotherapy treatment for inflammatory breast cancer

**Lost to follow-up after 3 months from surgical treatment

ND = not done

N/A = not applicable

DOD = Died of disease (breast cancer-specific death)

AWD = Alive with metastatic disease

NED = No evidence of disease
