# Supplementary material for: Patient-derived xenografts of triple-negative breast cancer reproduce molecular features of patient tumors and respond to mTOR inhibition
Source: Breast Cancer Res. 2014 Apr 7;16(2):R36. doi: 10.1186/bcr3640 (PMC4053092; doi:10.1186/bcr3640)
Supplement: Additional file 4: Figure S1 — Array CGH profiles of all chromosomes comparing patient (p) and xenograft (x) for samples SUTI 151 and SUTI 110. [file bcr3640-S4.pptx]

## Slide 1
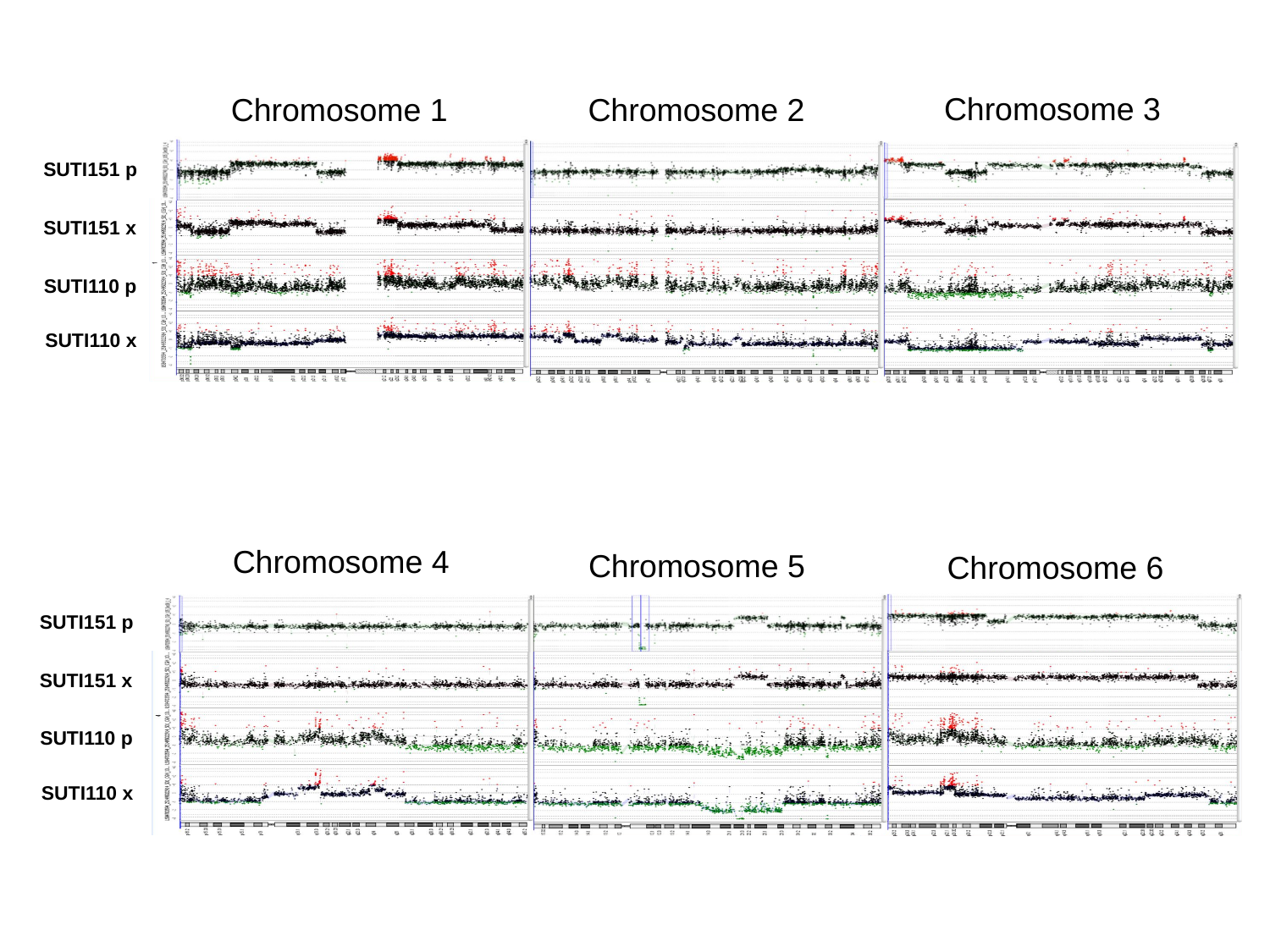

Chromosome 3
Chromosome 1
Chromosome 2
SUTI151 p
SUTI151 x
SUTI110 p
SUTI110 x
Chromosome 4
Chromosome 5
Chromosome 6
SUTI151 p
SUTI151 x
SUTI110 p
SUTI110 x

## Slide 2
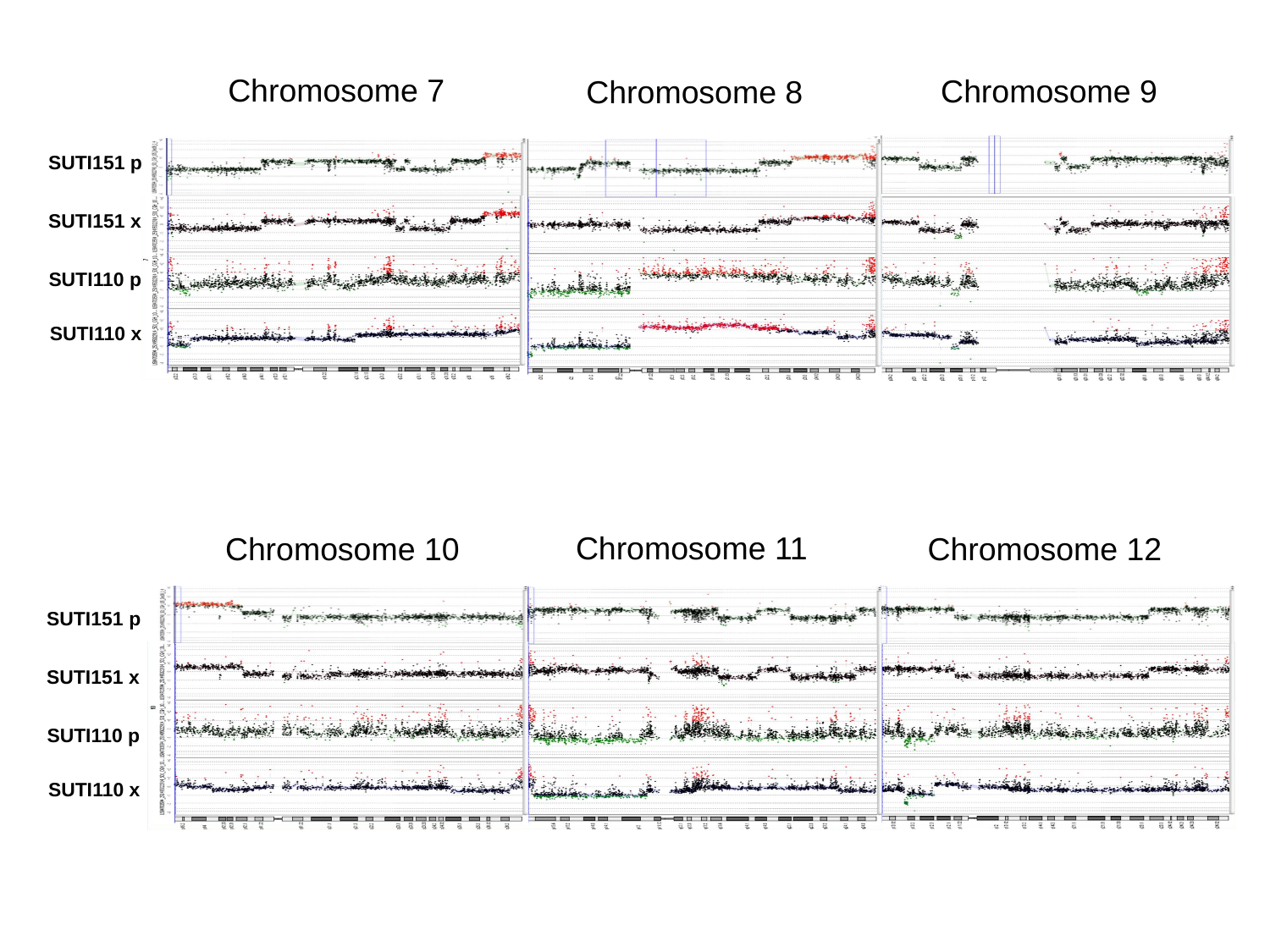

Chromosome 7
Chromosome 9
Chromosome 8
SUTI151 p
SUTI151 x
SUTI110 p
SUTI110 x
Chromosome 11
Chromosome 12
Chromosome 10
SUTI151 p
SUTI151 x
SUTI110 p
SUTI110 x

## Slide 3
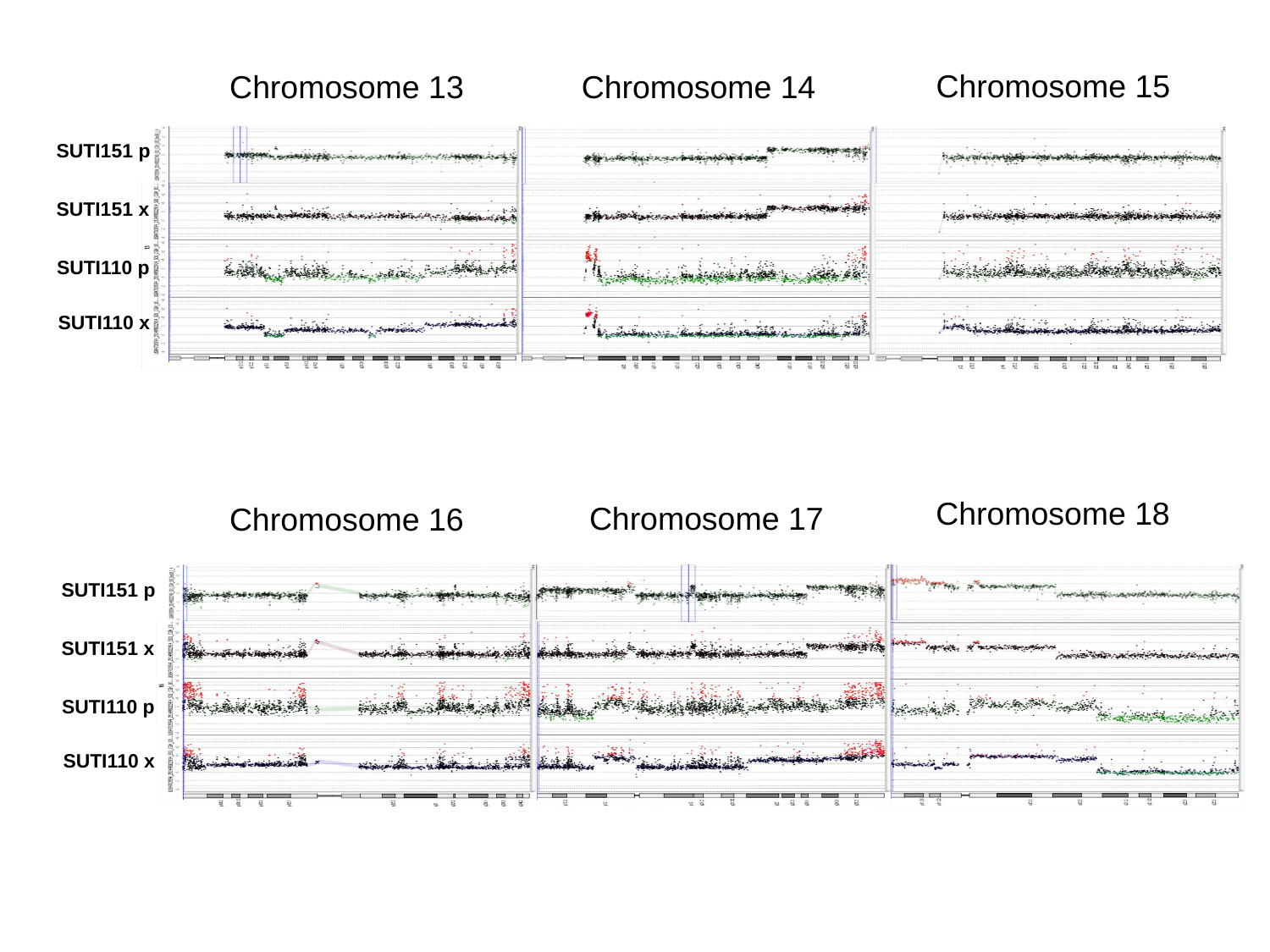

Chromosome 15
Chromosome 13
Chromosome 14
SUTI151 p
SUTI151 x
SUTI110 p
SUTI110 x
Chromosome 18
Chromosome 17
Chromosome 16
SUTI151 p
SUTI151 x
SUTI110 p
SUTI110 x

## Slide 4
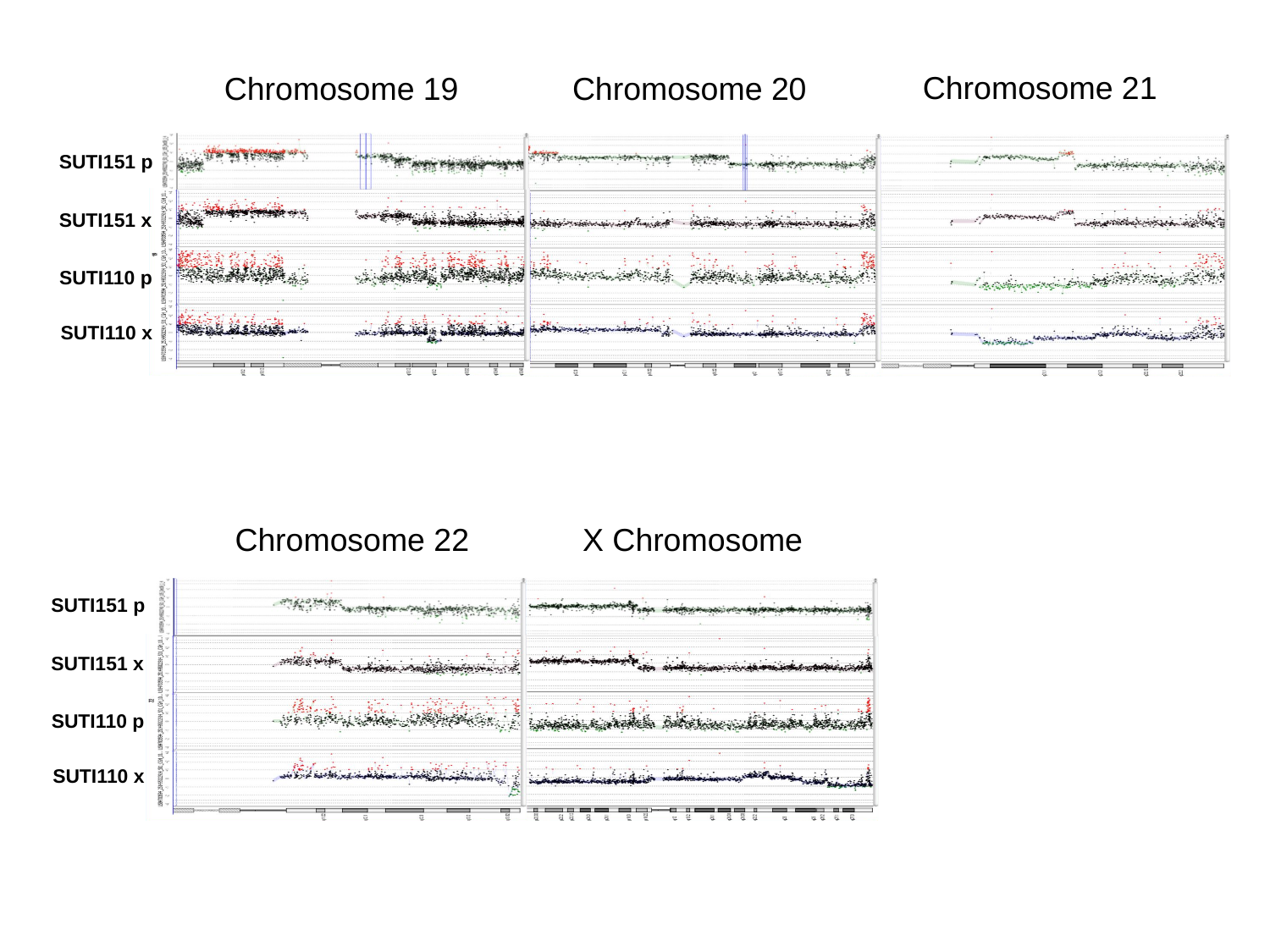

Chromosome 21
Chromosome 19
Chromosome 20
SUTI151 p
SUTI151 x
SUTI110 p
SUTI110 x
Chromosome 22
X Chromosome
SUTI151 p
SUTI151 x
SUTI110 p
SUTI110 x
