# Supplementary material for: Patient-derived xenografts of triple-negative breast cancer reproduce molecular features of patient tumors and respond to mTOR inhibition
Source: Breast Cancer Res. 2014 Apr 7;16(2):R36. doi: 10.1186/bcr3640 (PMC4053092; doi:10.1186/bcr3640)
Supplement: Additional file 6: Figure S2 — Validations of rapamycin response prediction. A. Plots of predicted rapamycin sensitivity of MDA-MB-468 cells based on GEO data set GSE18571. As indicated, MDA-MB-468 was treated with either vehicle control (DMSO) or rapamycin in both cell culture and xenografts. Xenograft tumors were collected after 1 day or 22 days of treatment. B. Plots of predicted sensitivity to rapamycin in Connectivity Map samples from nine independent batches. Samples are grouped as untreated controls (Untreated), rapamycin-treated (Rapamycin), PI3K inhibitors-treated (PI3K inhibitors), or treated with drugs other than rapamycin or PI3K inhibitors (Other drugs). The bar showed the mean of the predicted sensitivity with 1 as the highest and 0 the lowest predicted sensitivity to rapamycin. Figure S3 Correlation of actual sensitivity and predicted sensitivity. Correlation of actual sensitivity to rapamycin treatment (indicated by EC50) and predicted sensitivity by the rapamycin response signature of 18 breast cancer cell lines (scattered dots). A regression line was drawn to show the degree of correlation. [file bcr3640-S6.docx]

**Figure S2**


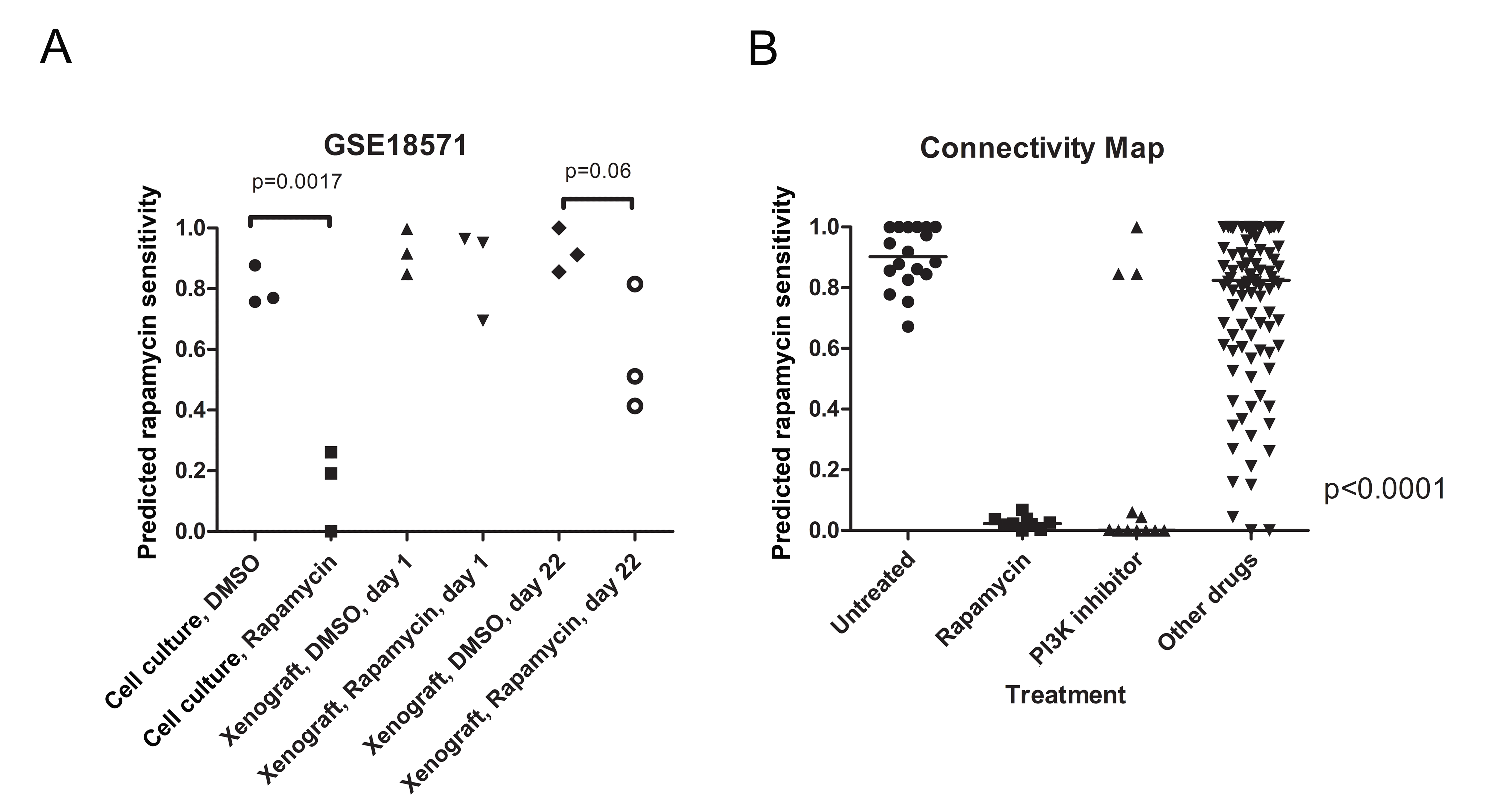


**Figure S2:** Validations of rapamycin response prediction. A. Plots of predicted rapamycin sensitivity of MDA-MB-468 cells based on GEO data set GSE18571. As indicated, MDA-MB-468 was treated with either vehicle control (DMSO) or rapamycin in both cell culture and xenografts. Xenograft tumors were collected after 1 day or 22 days of treatment. B. Plots of predicted sensitivity to rapamycin in Connectivity Map samples from nine independent batches. Samples are grouped as untreated controls (Untreated), rapamycin-treated (Rapamycin), PI3K inhibitors-treated (PI3K inhibitors), or treated with drugs other than rapamycin or PI3K inhibitors (Other drugs). The bar showed the mean of the predicted sensitivity with 1 as the highest and 0 the lowest predicted sensitivity to rapamycin.

**Figure S3**

**Figure S3:** Correlation of actual and predicted sensitivity. Correlation of actual sensitivity to rapamycin treatment (indicated by EC50) and predicted sensitivity by the rapamycin response signature of 18 breast cancer cell lines (scattered dots). A regression line was drawn to show the degree of correlation.
