# Supplementary material for: Patient-derived xenografts of triple-negative breast cancer reproduce molecular features of patient tumors and respond to mTOR inhibition
Source: Breast Cancer Res. 2014 Apr 7;16(2):R36. doi: 10.1186/bcr3640 (PMC4053092; doi:10.1186/bcr3640)
Supplement: Additional file 7: Table S5 — Phosphorylation levels of S6K1, 4EBP1, eIF4E and mTOR by immunoblot after rapamycin or CCI-779 treatment. [file bcr3640-S7.docx]

**Table S5: Phosphorylation levels of S6K1, 4EBP1, eIF4E and mTOR by immunoblot after rapamycin or CCI-779 treatment.**

|  | **Rapamycin treatment *** | | | |
| --- | --- | --- | --- | --- |
|  | **p-S6K1** | **p-4EBP1** | **p-eIF4E** | **p-mTOR** |
| **Metastatic tumor derived** |  |  |  |  |
| SUTI151M | 110 | 72 | 93 | 91 |
| **Primary tumor derived** |  |  |  |  |
| SUT097 | 65 | 36 | 18 | 30 |
| SUTI151 | 70 | 43 | 14 | 80 |
| SUTI319 | 67 | 108 | 6 | 78 |
| SUTI103 | 78 | 22 | 18 | 29 |
| SUTI110 | 68 | 19 | 52 | 59 |
| SUTI368 | 57 | 3 | 107 | 6 |
| **Average (%)** | **67** | **38** | **36** | **47** |
| **Decreased by (%)** | **33** | **62** | **64** | **53** |
|  |  |  |  |  |
|  | **CCI-779 treatment **** | | | |
|  | **p-S6K1** | **p-4EBP1** | **p-eIF4E** | **p-mTOR** |
| **Metastatic tumor derived** |  |  |  |  |
| SUTI151M | 109 | 87 | 70 | 125 |
| **Primary tumor derived** |  |  |  |  |
| SUT097 | 75 | 73 | 29 | 21 |
| SUTI151 | 41 | 28 | 108 | 114 |
| SUTI319 | 75 | 62 | 7 | 7 |
| **Average (%)** | **64** | **54** | **48** | **47** |
| **Decreased by (%)** | **36** | **46** | **52** | **53** |

***** Calculated by [rapamycin/(pretreatment+control)/2]*100 based on Western blot signal intensity.

******Calculated by [CCI-779/(pretreatment+control)/2]*100 based on Western blot signal intensity.
